# Alcohol-Induced Neuroinflammatory Response and Mitochondrial Dysfunction on Aging and Alzheimer’s Disease

**DOI:** 10.3389/fnbeh.2021.778456

**Published:** 2022-02-10

**Authors:** Brandon Emanuel León, Shinwoo Kang, Gabriela Franca-Solomon, Pei Shang, Doo-Sup Choi

**Affiliations:** ^1^Regenerative Sciences Program, Center for Regenerative Medicine, Mayo Clinic, Rochester, MN, United States; ^2^Department of Molecular Pharmacology and Experimental Therapeutics, Mayo Clinic, Rochester, MN, United States; ^3^Neuroscience Program, Mayo Clinic College of Medicine and Science, Rochester, MN, United States; ^4^Department of Psychiatry and Psychology, Mayo Clinic College of Medicine and Science, Rochester, MN, United States

**Keywords:** alcohol use disorder, mitochondria, morphology, aging, dementia, Alzheimer’s disease

## Abstract

Mitochondria are essential organelles central to various cellular functions such as energy production, metabolic pathways, signaling transduction, lipid biogenesis, and apoptosis. In the central nervous system, neurons depend on mitochondria for energy homeostasis to maintain optimal synaptic transmission and integrity. Deficiencies in mitochondrial function, including perturbations in energy homeostasis and mitochondrial dynamics, contribute to aging, and Alzheimer’s disease. Chronic and heavy alcohol use is associated with accelerated brain aging, and increased risk for dementia, especially Alzheimer’s disease. Furthermore, through neuroimmune responses, including pro-inflammatory cytokines, excessive alcohol use induces mitochondrial dysfunction. The direct and indirect alcohol-induced neuroimmune responses, including pro-inflammatory cytokines, are critical for the relationship between alcohol-induced mitochondrial dysfunction. In the brain, alcohol activates microglia and increases inflammatory mediators that can impair mitochondrial energy production, dynamics, and initiate cell death pathways. Also, alcohol-induced cytokines in the peripheral organs indirectly, but synergistically exacerbate alcohol’s effects on brain function. This review will provide recent and advanced findings focusing on how alcohol alters the aging process and aggravates Alzheimer’s disease with a focus on mitochondrial function. Finally, we will contextualize these findings to inform clinical and therapeutic approaches towards Alzheimer’s disease.

## Introduction

Alzheimer’s disease (AD) constitutes 60%–80% of dementia cases worldwide. AD results in serious deteriorations in memory and cognitive abilities that lead to loss of independence and quality of life. As of 2021, 6.2 million Americans over 65 are living with AD and 72% are 75 years or older (Alzheimer’s Association, 2021). Studies over the past 25 years have suggested that the prevalence and incidence of AD in the United States and other high-income Western countries have declined. However, as the number of individuals aged 65 and older increases, the number of people diagnosed with AD is expected to quadruple by 2050 with 43% of those individuals needing high levels of care (Brookmeyer et al., 2007). Furthermore, global projections indicate that the majority of the prevalence and burden will take place in low- and middle-income countries (Alzheimer’s Association, 2021). These projections highlight the need to further our understanding of AD pathology and develop effective therapies to offset the financial and emotional burden. AD pathology is classically characterized by intracellular tau tangles and extracellular amyloid-β (Aβ) plaques ostensibly causing cognitive deficits and neurodegeneration (Kamal et al., 2020). However, numerous clinical drug trials targeting Aβ have failed to yield much success opening the door for novel intervention targets (Huang et al., 2020) including neuroinflammation and mitochondrial dysfunction (Calsolaro and Edison, 2016; Tyumentsev et al., 2018).

The greatest risk factor for AD is aging, but binge and chronic alcohol abuse has been identified as a risk factor for AD. Interestingly, mild-moderate alcohol use has been consistently associated with decreased risk for AD and dementia (Luchsinger and Mayeux, 2004), thus, we will focus on the more hazardous binge and chronic alcohol abuse. Heavy and chronic alcohol abuse is associated with changes in brain structure, cognitive deficits, and decreased brain volume (Rehm et al., 2019). Alcohol use disorder (AUD) is defined as the impaired ability to control or stop alcohol consumption despite negative social, occupational, or health consequences (American Psychiatric Association, 2013). AUD is among the most prevalent substance use disorders and disproportionately afflicts men compared to women (Rehm and Shield, 2019). However, since 2001, the instances of AUD have increased among women, racial and ethnic minorities, the socioeconomically disadvantaged, and, relevant to our review, older adults (Grant et al., 2017). AUD and hazardous alcohol abuse may contribute to the risk for AD development through neuroinflammatory response and mitochondrial dysfunction. Neuroinflammation is a hallmark feature of AD (Gomez-Nicola and Boche, 2015; Liu et al., 2015) and alcohol exposure induces a neuroinflammatory response (Crews et al., 2017). In addition, mitochondrial function, vital to neuronal function, is consistently impaired in AD (Wang et al., 2020) and with alcohol exposure (Tapia-Rojas et al., 2019).

Mitochondria are central to several cellular processes including energy production, metabolic pathways, signal transduction, lipid biogenesis, and apoptosis (Araiso et al., 2019). Neurons are energetically demanding; thus, proper mitochondrial energy homeostasis is vital to sustaining appropriate neural activity. Through oxidative phosphorylation (OXPHOS) and other metabolic pathways, mitochondria provide the substrates necessary to sustain neural activity. In addition, mitochondria are highly dynamic organelles that adapt to cellular demands. Through cycles of fission and fusion, mitophagy selectively disposes of dysfunctional mitochondria while biogenesis generates new mitochondria from pre-existing mitochondria. Mitochondria are also trafficked to dendrites and axons allowing direct energy supply in these subcellular components (Li et al., 2004; Chen and Chan, 2009). Therefore, deficiencies in mitochondrial bioenergetics and dynamics have dire consequences for the survival and health of its host cell and contribute to the pathogenesis of neurodegenerative diseases, especially AD (Wang et al., 2020).

In this review, we will summarize recent findings on hazardous alcohol-induced neuroinflammatory response and age-related mitochondrial dysfunction. We will then contextualize these findings to evaluate the mechanisms by which alcohol-induced neuroinflammatory response and mitochondrial dysfunction may contribute to the onset and progression of AD. Finally, we will summarize developments in novel therapeutics targeting neuroinflammation and mitochondria for AUD and AD.

## Alcohol and Cell-Specific Neuroinflammatory Response

Alcohol is a known modulator of systemic immune signaling and perturbs the expression of several immune-related genes (Neupane, 2016). Alcohol increases gut permeability and bacterial translocation into the periphery (Adachi et al., 1995), induces an inflammatory response in the liver, and promotes the systemic release of pro-inflammatory cytokines (Crews et al., 2017). Additionally, circulating endotoxins may activate peripheral immune cells including monocytes, macrophages, T lymphocytes, and dendritic cells to further release pro-inflammatory cytokines and chemokines (Pascual et al., 2015). While the blood-brain barrier (BBB) maintains a relatively isolated environment from the body, systemic inflammation may cross over into the brain and induce neuroinflammation through three known routes: Vagal nerve afferents, pro-inflammatory factors crossing the BBB, and activated monocytes translocating into the CNS (Crews et al., 2017).

### Microglia

Microglia, resident macrophages in the CNS, are the primary mediators of the neuroimmune response and are implicated in the pathology of AUD and AD. Microglia express cytokine, chemokine, and pattern recognition receptors (PPRs) that induce the expression of pro- or anti-inflammatory mediators when activated (Erickson et al., 2019). Classically, ramified, or resting microglia surveil their environment through cellular projections in search of infection or injury whereupon microglia adopt distinct activation profiles, M1 and M2, which have pro and anti-inflammatory effects respectively. M1 microglia adopt an amoeboid-like morphology and secrete pro-inflammatory molecules including interleukin (IL)-1, IL-6, and tumor necrosis factor-alpha (TNF-α). In contrast, M2 microglia secrete anti-inflammatory molecules including transforming growth factor-beta (TGF-β) and IL-10 (Tang and Le, 2016). However, recent transcriptional studies point to more nuanced and complex microglial activation profiles (Xue et al., 2014) that are dependent on the type of injury, insult, or brain region (Wes et al., 2016; De Biase et al., 2017). For example, in an AD mouse model, a distinct subpopulation of microglia demonstrated upregulated lipid metabolism, upregulated phagocytic-related genes, and spatially clustered around Aβ deposits (Keren-Shaul et al., 2017).

Alcohol exposure sensitizes the activation of microglia through toll-like receptors (TLRs), purinergic P2X receptors (PRXR), and cytokine signaling (Crews et al., 2017). TLRs are activated by both pathogen-associated molecular patterns (PAMPs) and damage-associated molecular patterns (DAMPs). *In vitro*, a high dose of alcohol exposure induced increases in TLR4, TLR2, IL-1β, TNF- α, and inducible nitric oxide synthetase (iNOS) expression. This is accompanied by an enhanced generation of reactive oxygen species (ROS; Fernandez-Lizarbe et al., 2013). It is well established that alcohol binds to gamma-aminobutyric acid (GABA) A receptor (GABA_A_R; Mihic et al., 1997). Thus, it is possible that alcohol increases TLR4 and TLR2 expression through activation of GABA_A_R although the exact mechanism remains unknown. AUD patient brains with BBB disruptions displayed enhanced microglial activation indicated by amplified ionized calcium-binding adaptor molecule 1 (IBA1) immunoreactivity. These findings were paralleled in mice exposed to binge-like alcohol drinking while the increase in IBA1 immunoreactivity was attenuated by TLR4 knockout (Rubio-Araiz et al., 2017). Transcriptomic studies of 2 bottle choice mice further demonstrated network perturbations in TLR and TGF-β signaling in microglia isolated from the prefrontal cortex (McCarthy et al., 2018).

P2XRs are activated by extracellular adenosine triphosphate (ATP) and are heavily expressed in microglia. P2X7R activation results in the release of pro-inflammatory cytokines IL-1β and IL-18 (He et al., 2017) which is mediated by the activation of nucleotide oligomerization domain containing protein 1 (NOD)-, leucine-rich repeat (LRR)-, and pyrin domain containing protein 3 (NLRP3) inflammasome complex (Iwata et al., 2016). Interestingly, NLRP3 inflammasome activation is a known pathogenic inflammatory response that occurs in AD (Halle et al., 2008; Heneka et al., 2013) Alcohol exposure of 100 mM, comparable to toxic levels in humans, in murine BV2 microglia had differential effects on P2X4R and P2X7R activity. Alcohol inhibited P2X4R activation while sparing P2X7R channel activity. Alcohol reduced P2X4R-mediated microglial migration but potentiated P2X7R pore formation. Alcohol exposure also enhanced P2X7R mediated IL-1β secretion and increased protein expression of both P2XR subtypes (Asatryan et al., 2018). Alcohol is implicated to act as a negative allosteric modulator for P2X4Rs having high and low-affinity sites corresponding to low and high concentrations of alcohol, respectively. In support of this, ivermectin (IVM), a positive allosteric modulator of P2X4R, reduces ethanol intake and operant self-administration in rodents (Franklin et al., 2014).

Besides playing a reactive role, microglial recruitment and activation may also contribute to the development and progression of AUD and AD. Depletion of microglia using PLX5622, a colony-stimulating factor-1 receptor antagonist, prevented increases in alcohol consumption and decreased anxiety-like behavior during abstinence during an alcohol vapor two-bottle choice drinking paradigm. Microglial depletion also rescued expression changes in genes related to inflammation, glutamatergic neurotransmission, and GABAergic neurotransmission in the prefrontal cortex and central nucleus of the amygdala (Warden et al., 2020). In a model of acute binge drinking, PLX5622 mediated depletion of microglia also resulted in increased expression of anti-inflammatory genes (Walter and Crews, 2017). Minocycline, an antibiotic that reduces microglia activation, reduced alcohol intake and preference in the two-bottle choice drinking paradigm in mice (Agrawal et al., 2011), and decreased withdrawal-induced anxiety and relapse in rats (Gajbhiye et al., 2018). These studies indicate that pharmacologically targeting microglia could be an effective strategy to prevent the development of AUD and withdrawal symptoms following abstinence. For AD, recent work demonstrated A2AR/N-methyl d aspartate ionotropic glutamate receptors (NMDAR) complexes are upregulated in activated microglia in the hippocampus of an AD mouse model. In neurons, while there was no difference in the number of complexes, activation of adenosine A2AR resulted in higher NMDAR functionality indicated by intracellular cyclic adenosine monophosphate levels and extracellular signal-regulated kinases 1/2 phosphorylation. Through A2AR antagonism, it may be possible to reduce glutamatergic excitotoxicity (Franco et al., 2020) and microglial release of pro-inflammatory mediators (Saura et al., 2005). Overall, these studies suggest that pharmacological targeting of microglia could be a novel mechanism to address AUD and AD.

### Astrocytes

Astrocytes are involved in a myriad of physiological processes including maintaining appropriate ionic concentrations, supporting neurons bioenergetically, neurotransmitter clearance, bridging the BBB, and maintaining the integrity of synapses (Khakh and Sofroniew, 2015). Astrocytes are also a component of the immune system in the CNS. While astrocytes express chemokine and cytokine receptors, astrocytes partly depend on microglia for their activation. In response to LPS administration, microglial IL-1α, TNF, and complement component 1 subcomponent q (C1q) mediate A1 astrocytic activation. A1 astrocytes reduced neuronal survival, outgrowth, synaptogenesis, and were observed in post-mortem tissue from patients with neurodegenerative diseases including AD (Liddelow et al., 2017).

Astrocyte activities are essential for addictive behaviors (Scofield and Kalivas, 2014; Corkrum et al., 2020; Kang et al., 2020; Kang and Choi, 2021), especially for AUD (Ayers-Ringler et al., 2016; Erickson et al., 2018, 2021; Lindberg et al., 2018). Astrocytes regulate neuronal activities of glutamatergic neurons in the cortico-striatal and dopaminergic neurons in the ventral tegmental area (VTA)-nucleus accumbens (NAc) circuits (Scofield and Kalivas, 2014; Corkrum et al., 2020). Importantly, astrocyte-neuron interactions are reciprocal in regulating addictive behaviors (Durkee and Araque, 2019). Interestingly, astrocytes are also central mediators of adenosine signaling which is implicated in the pathology of AUD and neurodegenerative disorders (Kim et al., 2013). In the CNS, adenosine regulates neuronal activity by modulating the signaling of other neurotransmitters. For example, the adenosine receptors, A1 and A2A, often work in tandem dimerizing with each other and with dopamine and glutamate receptors to fine-tune dopaminergic and glutamatergic signaling. Astrocytes release ATP into the extracellular space, where ATP is converted to adenosine by ecto-nucleotidases. Astrocytes also release adenosine through bidirectional transporters especially equilibrative nucleoside transporter 1 (ENT1). ENT1 knockout (KO) mice display reduced ataxic and hypnotic responses to alcohol exposure and increased alcohol intake compared to wild-type littermates. This is associated with decreased adenosine tone on A1 receptors indirectly measured through decreased A1 receptor-mediated inhibition of glutamate excitatory postsynaptic currents in the nucleus accumbens (Choi et al., 2004). Furthermore, adenosine-mediated glutamatergic signaling reduces sensitivity to alcohol and may predispose individuals to AUD (Chen et al., 2010). In addition, pharmacological inhibition, and genetic deletion of ENT1 reduced the expression of glutamate transport 1 (GLT1) and astrocyte-specific aquaporin 4 (AQP4). Ceftriaxone administration, an antibiotic which is known to decrease alcohol consumption in rodents, elevated GLT1 and AQP4 expression in the striatum, and reduced alcohol consumption in mice indicating that adenosine signaling mediates GLT1 and AQP4 expression (Lee et al., 2013). ENT1 KO mice also displayed reduced glial fibrillary acidic protein (GFAP) expression in the dorsal striatum suggesting that ENT1 and adenosine regulate GFAP expression and astrocytic function (Hinton et al., 2014). The adenosine analog, N6-(4-hydroxybenzyl)-adenosine (NHBA), which activates A2AR and inhibits ENT1, reduced alcohol intake and alcohol reward seeking behavior in mice (Hong et al., 2019). Chronic alcohol exposure in mice also alters glucose and lactate concentrations in the brain and decreases the expression of monocarboxylate transporter indicating that alcohol disrupts bioenergetic homeostasis between neurons and glia (Lindberg et al., 2019). This suggests impaired alcohol-induced astrocytic-neuronal bioenergetic crosstalk may contribute to impaired energy metabolism seen in AD (Wang et al., 2020), and the important roles astrocytes and astrocytic signaling play in the development of AUD.

## Mitochondria and Neuroinflammation

**Figure 1:**
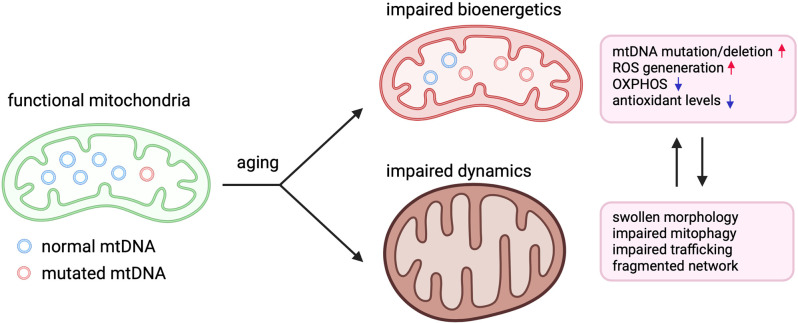
Perturbation of mitochondrial bioenergetics and dynamics in aging. Illustration demonstrating how aging negatively affects mitochondrial bioenergetics and dynamics in aging. In aged humans and animals, higher levels of mutations and deletions in mitochondrial DNA (mtDNA) lead to reduced mitochondrial protein expression and impairments in oxidative phosphorylation (OXPHOS), which exacerbates the generation of reactive oxygen species (ROS). Mitochondria also appear fragmented and swollen while also displaying impaired mitophagy and trafficking to neuronal compartments such dendrites and axons.

Mitochondrial are uniquely positioned as junctions of innate immunity by transducing immune signals, metabolic reprogramming, and generation of ROS and DAMPs (Figure 1). The outer mitochondrial membrane harbors mitochondrial antiviral signaling protein (MAVS) which signals the presence of cytosolic viral double-stranded RNA, leading to the activation of nuclear factor kappa B (NF-κB) and interferon regulator factor 3 (IRF3) to induce the expression of pro-inflammatory cytokines and type I interferon (IFN; Seth et al., 2005; Sun et al., 2006). Similarly, cytosolic DNA, detected by cyclic GMP-AMP synthase (cGAS), leads to the production of cyclic guanosine monophosphate-adenosine monophosphate (cGMP). cGMP binds to the stimulator of interferon genes (STING) located on the endoplasmic reticulum. STING then engages both IRF3 (Sun et al., 2013) and NF-κB to induce the expression of pro-inflammatory cytokines (Ishikawa and Barber, 2008).

Mitochondrial metabolic reprogramming is central to microglial activation. Activation triggers a metabolic shift from OXPHOS to glycolysis followed by the adoption of the M1 phenotype and pro-inflammatory response (Voloboueva et al., 2013; Gimeno-Bayón et al., 2014; Orihuela et al., 2016). The mitochondrial toxins 3-nitropropionic acid and rotenone prevented IL-4 induced phenotype transition to M2 associated with healing and anti-inflammatory response (Ferger et al., 2010). Furthermore, inhibition of complex I promoted activation of microglia and production of pro-inflammatory cytokines *via* induction of NF-κB and mitogen-activated protein kinases pathways (MAPKs; Ye et al., 2016). These studies implicate mitochondrial metabolic programming in microglial activation and suggest that perturbations in mitochondrial energy homeostasis may enhance pro-inflammatory microglial phenotypes associated with AUD and AD (Rose et al., 2017).

**Figure 2:**
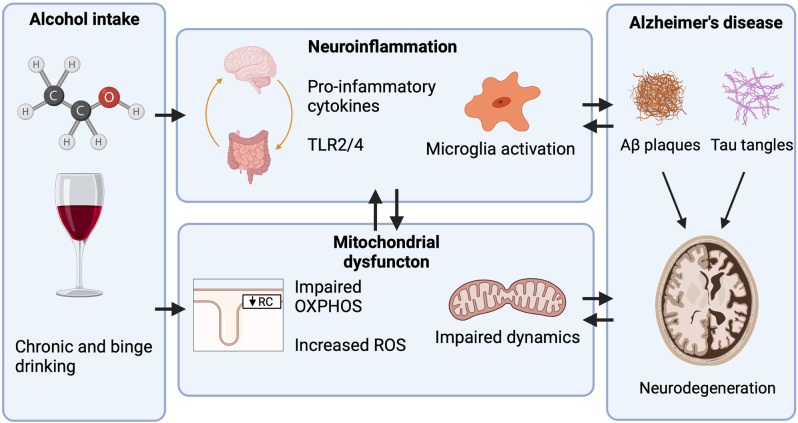
Mechanisms through which heavy alcohol abuse increases the risk for Alzheimer’s disease (AD). Only heavy alcohol abuse increases the risk for AD while light and moderate Alcohol use do not. Proposed mechanisms through which heavy alcohol abuse may increase the risk for AD. Alcohol increases peripheral inflammation by increasing the gut translocation of lipopolysaccharide (LPS) into circulation, where it activates peripheral immune cells and increases pro-inflammatory cytokine release. Pro-inflammatory cytokines then cross the blood-brain barrier (BBB) activating microglia and astrocytes. In the CNS, alcohol increases the expression of toll-like receptor 4 (TLR4) and High mobility group box 1 (HMGB1), a ligand for TLR4. TLR4 activation leads to NF-κB induction of pro-inflammatory cytokine release which further activates other microglia and astrocytes. Heavy alcohol abuse also impairs mitochondrial function by impairing OXPHOS, increasing the generation of ROS, and impairing mitochondrial dynamics. As neurons are energetically demanding cells, mitochondrial dysfunction is a contributing factor to neurodegeneration.

Mitochondria also generate ROS primarily through oxidative phosphorylation (OXPHOS) where the majority of ATP is produced (Figure 1). Normal physiological levels of ROS participate in cellular signaling (Shadel and Horvath, 2015) including the modulation of immune receptors (TLRs, NLRs, MAVS) and transcription factors (NF-κB and IRFs; Weinberg et al., 2015). However, overaccumulation of ROS and reactive nitrogen species (RNS), such as nitric oxide (NO), can damage both cellular and mitochondrial nucleic acid, proteins, and lipids. The balance between ROS production and neutralization is maintained by antioxidant systems. When these systems are compromised, ROS accumulation can result in the assembly of the NLRP3 inflammasome, damage to mitochondrial constituents, and neuroinflammation (Vezzani et al., 2020). ROS also activate microglia which further secrete ROS, RNS, and cytokines (McElroy et al., 2017). In addition, ROS-induced mitochondrial damage leads to deficiencies in OXPHOS. In the brain, where ATP is critically needed to maintain highly energetic processes such as neuronal potential, deficiencies in OXPHOS are a central facet to neuroinflammation and neurodegeneration (Vezzani et al., 2020; Figure 2). Furthermore, alcohol is known to increase ROS concentrations and decrease endogenous antioxidants. Mitochondria in the CNS are devoid of alcohol dehydrogenase and must metabolize alcohol *via* cytochrome P450 (CYP450) which metabolizes alcohol into acetaldehyde while producing superoxide anion (O^-2^) and hydrogen peroxide (H_2_O_2_; García-Suástegui et al., 2017). Preclinical studies have demonstrated that chronic alcohol exposure decreases glutathione and superoxide dismutase, antioxidants crucial to maintaining oxidative homeostasis. Alcohol administration increased NO, iNOS, and neuronal nitric oxide synthase levels (nNOS). Furthermore, the activities of mitochondrial complexes I, III, IV, Na+/K+ ATPase were also decreased, and brain mitochondria were sensitized to apoptotic stimuli (Almansa et al., 2009; Reddy et al., 2013). Acute administration of alcohol also increases linearized forms of mtDNA which were attenuated by antioxidant treatment (Mansouri et al., 2001).

Because of their prokaryotic origins, mitochondria are potent sources of DAMPs, and damage to their outer membrane, mitochondrial permeability transition, or cell death can result in the cytoplasmic and/or extracellular release of mitochondrial DAMPs (Grazioli and Pugin, 2018). Mitochondrial DAMPs include mitochondrial DNA (mtDNA), ATP, mitochondrial transcription factor A (TFAM), N-foryml peptides (NFPs), succinate, cardiolipin, and cytochrome C (Grazioli and Pugin, 2018). Like bacterial DNA, mtDNA is characterized by low levels of methylation but is protected by packing DNA into complexes known as nucleoids. Nucleoid packaging is mediated by TFAM which is a member of the high mobility group box family. Intratracheal injection mtDNA in mice promoted infiltration of CD68^+^ macrophages, production of pro-inflammatory cytokines (IL-1β, Il-6, and TNF-α), and activated p38 MAPK through TLR9 signaling (Gu et al., 2015). Interestingly, melatonin deficiency in mice lacking the N-acetyltransferase gene in cerebro-cortical neurons was associated with increased mtDNA release, decreased mitochondrial membrane potential, and increased oxidative stress. Cytosolic mtDNA induced activation of cGAS-STING-IRF3 pathway and resulted in inflammatory cytokine induction (Jauhari et al., 2020). Interestingly, melatonin is a known free radical scavenger (Zhang and Zhang, 2014) and is produced in the mitochondrial matrix (Suofu et al., 2017). mtDNA can also initiate the assembly of inflammasomes especially the NLRP3 and is absent in melanoma-2 (AIM2) inflammasomes. Assembly and activation of inflammasomes induce the expression of IL-1β and triggers caspase-1-dependent mitochondrial damage. Caspase-1 activity inhibits mitophagy, leads to enhanced ROS production, perturbs mitochondrial membrane potential, and results in mitochondrial permeabilization and network fragmentation (Shimada et al., 2012; Yu et al., 2014).

The principle pathologic feature of AD, Aβ, has been observed to accumulate within the mitochondria of human AD patients and mouse models of AD by interacting with Aβ peptide-binding alcohol dehydrogenase (ABAD; Lustbader et al., 2004). ABAD- Aβ interacts with several mitochondrial constituents to induce dysfunction. Blockage of ABAD-Aβ interaction increased enzyme activity of respiratory chain constituents, decreased oxidative stress, and improved spatial memory in transgenic mutant amyloid precursor protein (mAPP) mice (Yao et al., 2011). ABAD- Aβ interactions also potentiated cyclophilin D (CypD) mediated mitochondrial permeability transition and induced neuronal death, while knockout of CypD attenuated these effects and improved behavioral and synaptic function in mAPP mice (Du et al., 2008). In Tau22 mice, loss of NLRP3 inflammasome decreased tau hyperphosphorylation and intracerebral injection of Aβ containing brain homogenates induced tau pathology in NLRP3 dependent manner (Ising et al., 2019).

## Mitochondria, Energy Homeostasis, and Aging

Mitochondria are unique organelles due to their double membrane and own genome known as mitochondrial DNA (mtDNA). Besides rRNAs and tRNAs, mtDNA encodes for complexes I, III, IV, and V which are the essential constituents of the electron transport chain (ETC). Mitochondrial dysfunction is heavily implicated in the aging process and studies have demonstrated mitochondrial dysfunction occurs in the heart (Hoppel et al., 2017), muscles (Waltz et al., 2018), and livers (Ogrodnik et al., 2017) of older individuals. Interestingly, brains of older individuals displayed significant variation in the decline of OXPHOS suggesting that those with exacerbated declines in OXPHOS may be more prone to neuronal dysfunction (Ojaimi et al., 1999). In rat livers and spleens, the respiratory capacity of aged animals was significantly reduced compared to juveniles while there was no difference in the brain (Stocco and Hutson, 1978). Respiratory capacity also declines in the human liver, heart, and skeletal muscle tissue (Short et al., 2005). Mitochondrial respiratory chain complexes catalyze the oxidation of reducing equivalents, mainly nicotinamide adenine dinucleotide (NADH), using the terminal electron acceptor, oxygen, in the inner mitochondrial membrane (Genova and Lenaz, 2015). The activities of respiratory chain complexes I and IV decrease with age; however, the activities of complexes II, III, and IV remain unchanged (Navarro and Boveris, 2007). In a study in mice, protein levels of complexes I, II, IV, and V were increased in 12- and 18-month-old animals compared to 2-month-old animals. However, their mRNA expression in 24-month-old mice was decreased, indicating that age-related deficits in energy production are initially compensated by increased expression of ETC complexes. This compensation cannot be sustained for long and may be associated with the differences in the activity of ETC complexes with age (Manczak et al., 2005). Several decades of research have attempted to answer how age-related mitochondrial dysfunction occurs. The Free Radical Theory of Aging (MFRTA) and mtDNA insults (Chocron et al., 2019) have been proposed as mechanisms of age-related mitochondrial dysfunction. Importantly, the bioenergetics and dynamics of mitochondria are subject to extrinsic factors such as diet (Nisoli et al., 2005) and hormones (Cioffi et al., 2010) that may provide protection against oxidative damage and decelerate aging.

In contrast to nuclear DNA, mitochondria contain thousands of copies of mtDNA. Mutations and/or deletions of mtDNA can have deleterious effects on mitochondrial energy homeostasis. With age, wild-type and mutated mtDNA accumulate inside mitochondria and create a mosaic of functional and non-functional mtDNA. For mtDNA mutations to induce mitochondrial dysfunction, the ratio of mutated to wild type mtDNA must reach a threshold of 70%–90% (Rossignol et al., 2003). A study in healthy individuals demonstrated that low levels of mtDNA heteroplasmy are normal and are likely due to inheritance or single base substitutions (Payne et al., 2012). In women aged 17–85, those aged 70 years or older displayed increased mtDNA heteroplasmy (Zhang et al., 2017). In older men and women, high levels of mtDNA mutations in blood leukocytes were associated with reduced strength, cognitive abilities, and metabolic and cardiovascular function. Furthermore, participants with the highest levels of mutation load were at greater risk for developing dementia and stroke mortality (Tranah et al., 2018). Additionally, mutations in mtDNA are associated with an increased risk of cognitive decline and dementia (Kauppila et al., 2017). These studies suggest that reaching the 70%–90% threshold typically requires a lifetime to accumulate in enough cells but can cause bioenergetic deficiencies and are associated with aging phenotypes. The question remains whether mutation load correlates with age or drives aging phenotypes. Experimentally, the proofreading-deficient mtDNA polymerase (POLγ) mouse model provides interesting evidence demonstrating that abnormal accumulation of mtDNA mutations leads to reduced lifespan and aging-related phenotypes such as alopecia and osteoporosis. Briefly, the proofreading deficient POLγ leads to excessive accumulations of mtDNA mutations (Trifunovic et al., 2004). mtDNA mutations in these mice were not associated with signs of oxidative stress but displayed induction of apoptotic markers (Kujoth et al., 2005). Interestingly, offspring of mutator POLγ and wild type POLγ mice displayed mild signs of aging while offspring of mutator POLγ mice displayed severe brain malformations. These studies indicate that both inherited and somatic mtDNA mutations promote aging phenotypes (Ross et al., 2013). However, the levels of mtDNA mutations are much higher in these mice compared to aged controls and may not faithfully reflect what occurs in normal mice or humans. In contrast to mutations, mtDNA deletions are seldom inherited (Chinnery et al., 2004). Accumulation of mtDNA deletions has been reported in the brain (Corral-Debrinski et al., 1992; Taylor et al., 2014), muscle (Fayet et al., 2002), liver (Yen et al., 1991), and heart tissue of aged humans (Cortopassi and Arnheim, 1990). Additionally, Parkinson’s patients and age-matched controls displayed higher levels of mtDNA deletions in the substania nigra, especially in cells with respiratory chain deficiency (Bender et al., 2006, 2008; Kraytsberg et al., 2006). mtDNA deletions tend to be low in tissue homogenates; however, in individual cells, mtDNA deletions can be high where randomly distributed cells display respiratory chain deficiency (Baris et al., 2015). For example, muscle fibers of rats with electron transport chain deficiencies and atrophy displayed greater levels of deletions (Cao et al., 2001; Wanagat et al., 2001). Analogous to the mutator mouse model of mtDNA mutations, a mouse model expressing an inducible mitochondrially targeted restriction endonuclease demonstrated reduced levels of mtDNA encoded subunits of the electron transport chain and reduced oxidative phosphorylation activity (Fukui and Moraes, 2009). While the mechanisms by which mutations and deletions accumulate in mitochondria are still unclear, the wealth of studies has demonstrated that both mutations and deletions of mtDNA contribute to the aging phenotype.

Mitochondria are the primary sources of ROS (Figure 2). Respiratory chain complex activity results in the production of ROS as byproducts. Normal levels of ROS take part in physiological signal transduction such as regulating neural stem cell proliferation, self-renew, and neurogenesis (Le Belle et al., 2011). Hydrogen peroxide also modulates NF-κB by either stimulating or inhibiting NF-κB signaling depending on the context (Oliveira-Marques et al., 2009). To protect the cell and mitochondrial constituents, mitochondria deploy several ROS antioxidants that are adept at neutralizing ROS before oxidative damage can occur (Alexeyev, 2009). Several studies across different species have genetically overexpressed or knocked out antioxidant genes although no clear correlation was found between oxidant damage and lifespan (Bratic and Larsson, 2013). In *Drosophila*, for example, targeting catalase to the mitochondria nor overexpression of superoxide dismutase, catalase, and thioredoxin reductase extended lifespan (Mockett et al., 2003, 2010; Orr et al., 2003). Mice lacking superoxide dismutase and glutathione peroxidase-1 displayed increased DNA and protein oxidation without reduction of lifespan (Zhang et al., 2009). In *C. elegans*, the genetic deletion of superoxide dismutase yielded no obvious effects or extended lifespan (Doonan et al., 2008; Van Raamsdonk and Hekimi, 2009). These studies indicate that oxidative stress may not be the primary cause of aging. Nevertheless, under pathological conditions such as neurodegeneration or chronic heavy alcohol abuse, excessive oxidative stress may damage the molecular constituents of the brain. For example, the brain is highly concentrated with phospholipids and is vulnerable to lipid peroxidation (Kim et al., 2015). Excessive oxidative damage may also damage proteins and DNA which can induce mitochondrial dysfunction and cell death (García-Suástegui et al., 2017). Brains of older individuals and those diagnosed with neurodegenerative diseases displayed increased protein nitration and oxidation; decreased activity of antioxidant enzymes superoxide dismutase (SOD), catalase, and glutathione (GSH) reductase; and decreased complex I activity (Venkateshappa et al., 2012a, b). The peroxidation of lipids by ROS leads to signaling cascades that induce apoptosis and autophagy (Su et al., 2019).

Interestingly, increased ROS concentrations reduce the calcium concentration needed to open the mitochondrial permeability transition pore (Panel et al., 2018). The mitochondrial permeability transition has recently been proposed as a mechanism of aging and may contribute to the onset of neuronal injury in neurodegenerative disorders like AD (Figure 2). Briefly, the mitochondrial permeability transition is a sudden increase in the permeability of the inner mitochondrial membrane through a complexed nonspecific pore called the mitochondrial permeability transition pore (mPTP). The opening of the mPTP results in organelle swelling, drop in membrane potential, loss of mitochondrial substrates, and uncoupling of OXPHOS. The mPTP forms in response to increased matrix calcium levels and is sensitized by ROS, adenine nucleotide depletion, drop in membrane potential, and increased phosphate concentrations (Panel et al., 2018). In aged whole rat brains, mitochondrial susceptibility to mPTP opening was associated with impaired mitochondrial respiration and mitochondrial transmembrane electric potential (Marques-Aleixo et al., 2012; Krestinina et al., 2015); however, age-related mitochondrial impairments are not consistent in every brain region. For example, in aged Fischer 344 rats, striatal mitochondria were more sensitive to Ca^2+^ induced mPTP opening compared to cortical mitochondrial up to 24 months (LaFrance et al., 2005). Susceptibility to permeability transition may depend on CypD levels and neuron cell type. CypD has been implicated in the assembly of the mPTP. Interestingly, higher levels of cyclophilin D have been reported in neurons expressing glutamate decarboxylase or calbindin D-28k, GABAergic interneurons (Brustovetsky et al., 2003). Considering that these neurons are fast-spiking and energy demanding, brain regions with greater proportions of interneurons could explain the heterogenic susceptibility to Ca^2+^-induced mPTP formation (McCasland and Hibbard, 1997; Attwell and Laughlin, 2001; Brustovetsky et al., 2003).

## Alcohol, Mitochondria, and Alzheimer’S Disease

Mitochondrial dynamics sustain cellular integrity and control the disposal of aberrant constituents in the mitochondrial network (Kim et al., 2007; Youle and Narendra, 2011). Alcohol is known to substantially alter mitochondrial morphology in a concentration-dependent manner. For example, mitochondria demonstrated morphological changes and metabolic dysfunction after alcohol exposure (Suen et al., 2008; Flores-Bellver et al., 2014). In addition, mitochondrial fission protein Drp1 levels are significantly elevated after alcohol treatment in a concentration-dependent manner (Bonet-Ponce et al., 2015). Our own studies have demonstrated both morphological and functional changes in mitochondria in the prefrontal cortex in response to alcohol exposure (Shang et al., 2020). Distinct types of fission have been observed that determine the fate of mitochondria. Division at the midzone leads to proliferation of mitochondria, while division at the periphery leads to mitophagy (Kleele et al., 2021) During fission, fragmented mitochondria release cytochrome c and other intermembrane proteins that affect neighboring mitochondria and induce apoptosis (Detmer and Chan, 2007). Proteomics studies have demonstrated that 40 mitochondrial proteins, including key enzymes involved in β-oxidation, the tricarboxylic acid cycle, and amino acid metabolism, were altered after alcohol consumption (Venkatraman et al., 2004).

Alcohol-induced mitochondrial energy homeostasis dysfunction may alter the plasticity and intrinsic excitability of neurons depending on the age, dose, duration of exposure, and brain region. In the cerebral cortex, for example, in PD6 to PD9 rat pups, alcohol induced a higher incidence of potentiation, while PD13 and PD15 rat pups displayed greater attenuation. These changes in the intrinsic excitability may be attributed to altered Ca^2+^ dynamics in mitochondria (Harrison et al., 2017; Cannady et al., 2018). For instance, selective inhibitors of Ca^2+^ uptake and release blocked post tetanic potentiation while endoplasmic reticulum Ca^2+^ pump inhibitors and activators showed no effects (Tang and Zucker, 1997). Synaptic plasticity may also depend on mitochondrial respiratory capacity. The complex I inhibitor, rotenone, significantly impaired long-term potential in hippocampal neurons of rats (Kimura et al., 2012). Furthermore, selective inhibition of mitochondrial dihydroorotate dehydrogenase reduced the respiratory capacity of mitochondria and attenuated the firing rates of hippocampal neurons (Styr et al., 2019). Compared with neurons in the prefrontal cortex and hippocampus, motor neurons in the brainstem and hypoglossal nucleus are more sensitive to changes in calcium load and hypoxia, especially during complex IV inhibition (Bergmann and Keller, 2004). During alcohol withdrawal, increased neuronal excitability was found in the infralimbic region of the medial prefrontal cortex in mice chronically exposed to alcohol vapor (Pleil et al., 2015). Therefore, depending on the factors mentioned above, alcohol differentially affects the intrinsic excitability and plasticity of neurons (Tu et al., 2007; Weitlauf and Woodward, 2008).

Harwood et al. (2010) demonstrated the correlation between alcohol consumption and elevated risk of AD. Reducing hazardous alcohol intake increases the quality of life, cognitive ability, and longevity (Venkataraman et al., 2017). Alcohol can directly cross the BBB (Szabo and Lippai, 2014) and thus, chronic hazardous alcohol use can damage the nervous system especially the cortex and hippocampus (Harper et al., 1985; de la Monte and Kril, 2014). In differentiated neuroblastoma cells, chronic alcohol exposure in the range of 125–500 mg/dl dose-dependently increased tau protein levels and decreased cell viability. Chronic alcohol also increased beta secretase-1 (BACE1) *via* the production of ROS, suggesting that it causes neuronal damage and death induced by Aβ (Gendron et al., 2008). Cyclin-dependent kinase 5 (Cdk-5) induces oxidative stress by inactivating peroxiredoxin-I (Prx-I) and peroxiredoxin-II (Prx-II), the cytoplasmic peroxidase enzymes that process ROS. These results show that chronic alcohol might trigger increased ROS due to Cdk-5 (Liu et al., 2016). In addition, excessive alcohol intake increases p25, increased p25 proceeds to the neurotoxic pathway through Cdk5 (Kusakawa et al., 2000). Increases in Cdk5 and glycogen synthase kinase-3 β (GSK3-β) results in tau hyperphosphorylation-induced cognitive decline (Camp et al., 2006). A recent study demonstrated binge alcohol exposure leads to GSK3β activation which results in neurodegeneration, indicated by reduced NeuN positive neurons and ultrastructural analysis, and impaired spatial learning and memory in the Morris water maze (Ji et al., 2018). GSK3β activity is inhibited by phosphorylation in serine 9 (Ser9) while alcohol exposure leads to dephosphorylation at Ser9. *In vitro*, overexpression of GSK3β resulted in cellular sensitivity to 400 mg/dl alcohol exposure *via* induction of caspase 3 ultimately resulting in apoptosis (Liu et al., 2009). These studies indicate that GSK3β may act as a mediator bridging hazardous alcohol abuse, neurodegeneration, and cognitive deficits.

## Discussion

The increasing number of individuals diagnosed with AUD and AD highlights the need for novel therapeutics as many AD clinical trials fail to yield optimistic results (Huang et al., 2020). As discussed above, chronic neuroinflammatory response and mitochondrial dysfunction mechanistically link AUD and AD pathology and may be an avenue through which these disorders are addressed. Several anti-inflammatory and mitochondria targeting drugs are being developed and tested which are discussed in detail (Table 1). CP2 is a mitochondrial complex I inhibitor and AMP-activated protein kinase (AMPK) activator that improves mitochondrial bioenergetics and axonal trafficking, reduces inflammation and Aβ pathology, and improves synaptic activity (Zhang et al., 2015, 2019; Ray et al., 2018; Hara et al., 2019; Stojakovic et al., 2021). However, CP2 has not undergone clinical trials for AD. Additionally, CP2 has not been evaluated in the context of AUD. Minocycline is a tetracycline antibiotic that, like other antibiotics, reduces drinking in rodent models of AUD (Agrawal et al., 2011). Minocycline is thought to reduce drinking by inhibiting microglia activation (Regen et al., 2015; Clemens et al., 2018) and reducing levels of pro-inflammatory cytokines (Garcez et al., 2017). Interestingly, minocycline administrations improved spatial memory and provide protection against neuronal cell death in animal models of AD (Choi et al., 2007; Réus et al., 2015; Garcez et al., 2017). However, a clinical trial in which individuals with mild AD were treated with minocycline for over 2 years found no delay in the progress of cognitive or functional impairment (Howard et al., 2020). Phosphodiesterase 4 (PDE4) inhibitors are another drug class being investigated for AD and AUD. PDE4 inhibitor administrations reduce alcohol intake and preference in mice and rats (Blednov et al., 2014; Bell et al., 2015; Howard et al., 2020). Ibudilast also prevented Aβ1–42 induced memory impairments and reduced neuroinflammation and apoptotic responses (Wang et al., 2014). Ibudilast has been investigated in clinical trials for multiple sclerosis without any significant effects (Wang et al., 2014; Fox et al., 2021; Goodman et al., 2021). A promising novel Aβ antibody drug, aducanumab, received FDA approval under the accelerated approval pathway. Chronic administrations in aducanumab in Tg2576 mice rescued calcium homeostasis (Kastanenka et al., 2016). Aducanumab also demonstrated efficacy to reduce soluble and insoluble Aβ in transgenic mouse models of AD and patients with prodromal or mild AD. In patients with prodromal or mild AD, aducanumab also slowed cognitive decline in a phase 1b clinical trial (Sevigny et al., 2016). While promising, larger phase three trials, EMERGE and ENGAGE, initially failed to provide significant benefits from aducanumab. However, upon *post hoc* analysis, it was determined that there was sufficient clinical efficacy in a subset of participants. This has led to contention as many believe there is not enough evidence to conclude aducanumab is sufficiently efficacious (Howard and Liu, 2020; Schneider, 2020; Knopman et al., 2021). Finally, non-steroidal anti-inflammatory drugs (NSAIDs) are being tested as preventative therapies to slow the onset of AD. Specifically, cyclooxygenase-2 inhibitors (Zarghi and Arfaei, 2011) have demonstrated therapeutic effects in pre-clinical models of AD (Lim et al., 2000, 2001; Kotilinek et al., 2008). Chronic use of NSAIDs has demonstrated some benefit in prodromal stages of AD; however, their side effects pose concerns for widespread use (Imbimbo et al., 2010). More studies and investigations are needed to completely understand the underlying mechanisms through which these drugs exert improve mitochondrial function and anti-inflammatory properties. Realistically, a combination of therapies that target Aβ and Tau pathologies, chronic neuroinflammation, and mitochondrial dysfunction may be more effective than any of these alone.

**Table 1:** Potential anti-inflammatory medications for alcohol use disorder and Alzheimer’s disease.

Drug	Mechanism of action	Preclinical studies	Clinical trials
Minocycline	Inhibition of microglia activation (Regen et al., 2015; Clemens et al., 2018)	Reduces ethanol drinking in C57BL/6J mice (Agrawal et al., 2011) Reduces levels of IL-1β, TNF-α, IL-4, and IL-10 in mouse models of familial AD (Garcez et al., 2017) Improved spatial memory in radial arm maze in Aβ (1–42) administered mice (Garcez et al., 2017) Protects against oxidative damage/regulates energy metabolism in specific brain areas in model of chronic stress (Réus et al., 2015) Attenuated neuronal cell death and improved learning and memory in Aβ peptide_1#x02013;42_-infused rats (Choi et al., 2007)	544 participants of both sexes with mild AD were given 400 mg, 200 mg, and placebo over 2 years and found no delay in progress of cognitive or functional impairment (Howard et al., 2020)
Ibudilast, Mesopram, Rolipram, CDP 480	Phosphodiesterase 4 inhibitors	Reduced ethanol intake in in C57BL/6J mice (Blednov et al., 2014) Reduced ethanol intake in alcohol-preferring rats (Bell et al., 2015) Prevented Aβ_1–42_ induced memory impairments in morris water maze and attenuated neuroinflammatory and apoptotic responses (Wang et al., 2014)	Ibudilast 100 mg/kg in progressive multiple sclerosis (MS) patients did not reduce serum nor cerebrospinal fluid neurofilament light chain levels (Fox et al., 2021) Ibudilast did not attenuate focal inflammation pathology in progressive MS but had neuroprotective effect (Goodman et al., 2021)
Non-steroidal anti-inflammatory drugs (NSAIDs)	Cyclooxygenase-2 inhibitors (Zarghi and Arfaei, 2011)	Ibuprofen, naproxen, and MF-tricyclic rescued memory function in Aβ 42 overexpressing Tg2576 mice which was inversely associated with prostaglandin E2 levels (Kotilinek et al., 2008) In Amyloid precursor protein (APP) females, ibuprofen treatment reduced caspase activation per plaque and reduced Aβ levels (Lim et al., 2001) In APP mice, ibuprofen reduced cytokine levels, GFAP levels, and Aβ deposits (Lim et al., 2000)	Chronic use of NSAIDs prior to MCI or AD may be beneficial while use after Aβ deposition has already started may not be effective (Imbimbo et al., 2010)

Finally, it is critical to better elucidate the molecular mechanisms underlying the alcohol-induced neuroinflammatory responses, which may impair mitochondrial function in AD. A clear understanding of these processes may yield novel therapeutic targets to mitigate the severity of AD symptoms associated with hazardous alcohol drinking.

## Author Contributions

BEL, SK, GF-S, PS, and D-SC wrote the manuscript. BEL and D-SC prepared figures. All authors contributed to the article and approved the submitted version.

## Conflict of Interest

D-SC is a scientific advisory board member to Peptron Inc. Peptron Inc had no role in the preparation, review, or approval of the manuscript; nor the decision to submit the manuscript for publication. The remaining authors declare that the research was conducted in the absence of any commercial or financial relationships that could be construed as a potential conflict of interest.

## Publisher’s Note

All claims expressed in this article are solely those of the authors and do not necessarily represent those of their affiliated organizations, or those of the publisher, the editors and the reviewers. Any product that may be evaluated in this article, or claim that may be made by its manufacturer, is not guaranteed or endorsed by the publisher.
